# Selection on oxidative phosphorylation and ribosomal structure as a multigenerational response to ocean acidification in the common copepod *Pseudocalanus acuspes*

**DOI:** 10.1111/eva.12335

**Published:** 2015-11-23

**Authors:** Pierre De Wit, Sam Dupont, Peter Thor

**Affiliations:** ^1^ Department of Marine SciencesUniversity of GothenburgStrömstadSweden; ^2^ Department of Biological and Environmental SciencesUniversity of GothenburgFiskebäckskilSweden; ^3^ FramcentreNorwegian Polar InstituteTromsøNorway

**Keywords:** acclimation, adaptation, evolution, gene expression, ocean acidification, *Pseudocalanus*, transcription, transgenerational effects, translation

## Abstract

Ocean acidification is expected to have dramatic impacts on oceanic ecosystems, yet surprisingly few studies currently examine long‐term adaptive and plastic responses of marine invertebrates to *p*CO_2_ stress. Here, we exposed populations of the common copepod *Pseudocalanus acuspes* to three *p*CO_2_ regimes (400, 900, and 1550 μatm) for two generations, after which we conducted a reciprocal transplant experiment. A *de novo* transcriptome was assembled, annotated, and gene expression data revealed that genes involved in RNA transcription were strongly down‐regulated in populations with long‐term exposure to a high *p*CO_2_ environment, even after transplantation back to control levels. In addition, 747 000 SNPs were identified, out of which 1513 showed consistent changes in nucleotide frequency between replicates of control and high *p*CO_2_ populations. Functions involving RNA transcription and ribosomal function, as well as ion transport and oxidative phosphorylation, were highly overrepresented. We thus conclude that *p*CO_2_ stress appears to impose selection in copepods on RNA synthesis and translation, possibly modulated by helicase expression. Using a physiological hypothesis‐testing strategy to mine gene expression data, we herein increase the power to detect cellular targets of ocean acidification. This novel approach seems promising for future studies of effects of environmental changes in ecologically important nonmodel organisms.

## Introduction

Anthropogenic emissions of CO_2_ have increased the global *p*CO_2_ from 280 ppm at pre‐industrial times to the present day 400 ppm (IPCC 2013). About one‐third of emitted CO_2_ is absorbed by the world's oceans (Sabine et al. 2004). Dissolution of CO_2_ into surface water forms H_2_CO_3_, carbonic acid, which quickly dissociates into bicarbonate (HCO3^−^) ions as well as hydrogen (H^+^) ions, lowering the seawater pH (ocean acidification, OA) (Doney et al. 2009). These changes, which are predicted to persist for thousands of years to come, will expose marine animals to dramatically changed chemical conditions, and negative effects are predicted for many species and ecosystems (Wittman and Pörtner 2013).

Calanoid copepods constitute approximately 80% of the global zooplankton biomass (Mauchline 1998). Many fish species depend on copepods for prey during their larval life (Last 1980), and fish stock recruitments can vary closely with copepod biomass (Beaugrand et al. 2003; Castonguay et al. 2008). *Pseudocalanus* is widely distributed in temperate and Arctic seas (Aarbakke et al. 2011). They are intensely preyed upon (Ohman 1986; Thor et al. 2008), may at times contribute more than 1/3 of the total zooplankton biomass in boreal and Arctic waters (Lischka and Hagen 2005; Thor et al. 2005), and thus constitute important prey items for many fish species.

Tolerance of calanoid copepods to OA has been assessed in a number of studies to date, and many have found them to be remarkably resilient (Weydmann et al. 2012; Pedersen et al. 2013). However, most studies have focused on species such as *Calanus finmarchius* (Mayor et al. 2007, 2012; Hildebrandt et al. 2014; Pedersen et al. 2014b), which undergo seasonal diapause (metabolic depression) during which extracellular pH can drop to pH 5 (Schruender et al. 2013). So, these species could already be adapted to tolerate exposure to low pH conditions. Other studies have shown sensitivity even in species otherwise known to be resilient to large changes in water chemistry (Calliari et al. 2008; Cripps et al. 2014). Also, importantly, most are short‐term studies (e.g., Kurihara et al. 2004; Watanabe et al. 2006; Lewis et al. 2013; Engström‐Öst et al. 2014), mostly on adult females (Cripps et al. 2014), so they do not assess transgenerational plasticity or adaptation to low pH (but see Kurihara and Ishimatsu 2008; Pedersen et al. 2014a). Thor and Dupont (2015) recently conducted a multigeneration study, finding negative effects of short‐term (three weeks) pH stress on fecundity and metabolism in *Pseudocalanus acuspes*. These effects did not decrease after two generations in elevated *p*CO_2_ within the present range of natural variability (900 μatm), but in a higher *p*CO_2_ deviating from the present range of variability (1550 μatm), authors observed a transgenerational buffering effect decreasing negative effects to only half of those observed in acute pH stress treatments. Reciprocal transplant tests showed that this buffering was caused either by transgenerational plasticity (e.g., epigenetic changes in gene expression) or adaptive evolution, or a combination of both (Thor and Dupont 2015).

The actual mechanism by which OA affects marine organisms has been studied on several different levels. On the whole‐organism level, energy budgets are critical to consider, as in affected animals a greater fraction of the energy budget may be diverted to costs for maintenance, repair, and homeostasis (Pörtner et al. 2004; Stumpp et al. 2012a; Stumpp et al. 2013). On a cellular level, maintaining homeostasis is of utmost importance for cellular function, including mitochondrial function through the electron transport chain (Cortassa et al. 2009), protein folding (Dobson 2003), and cytoskeleton organization (Squirrell et al. 2001). Effects of low pH could lead to increased energy demand for proton pump action maintaining homeostasis (Stumpp et al. 2012b; Pan et al. 2015; Jager et al. 2016). Thus, it could be beneficial for cells to be able to down‐regulate certain functions (e.g., cell division) in order to make more energy available for core function such as maintenance of homeostasis, the end result being slower growth but a higher ability to function in a stressful environment (Stumpp et al. 2011).

At the molecular level, two processes allow for buffering of negative impacts of pH stress: acclimation and adaptation (Calosi et al. 2013; Reusch 2014). Acclimation can be a short‐term, reversible process within an individual, or a transgenerational development of different reaction norms due to for example maternal effects (e.g., egg quality) or epigenetic changes affecting gene expression responses to stress (Riebesell and Gattuso 2014; Magozzi and Calosi 2015). Adaptation on the other hand is a slower process that depends on heritable genetic variation in traits associated with tolerance for natural selection to act upon (Munday et al. 2013; Stillman and Paganini 2015). In the event of rapid environmental change, adaptation from standing genetic variation allows for rapid response (Hermisson and Pennings 2005). This is likely to occur in large populations that experience long‐term environmental fluctuations on a regular basis and has been shown to be common in the marine environment (Johannesson et al. 2010; Feulner et al. 2013; Pespeni et al. 2013; De Wit et al. 2014; Gosset et al. 2014). In many cases, this genetic variation exist as low‐frequency alleles that are neutral (or nearly neutral) in the background environment, but as they become adaptive they can quickly increase in frequency over a few generations, allowing the population to evolve their tolerance limit beyond that possible by nongenetic change (i.e., acclimation). In the most beneficial of cases, it has been hypothesized that a combination of nongenetic short‐term changes can combine with longer‐term genetic changes to facilitate evolution of tolerance limits (Stillman 2003; Ghalambor et al. 2007; Sunday et al. 2014).

This study is an examination of the molecular response of the copepod *P. acuspes* used in the experimental setup of Thor and Dupont (2015). In short, copepods were kept for two generations in one of three different *p*CO_2_ environments and then reciprocally transplanted. These reciprocal transplants tests indicated that while observed changes in fecundity were caused solely by phenotypic plasticity (i.e., acclimation) at the intermediate *p*CO_2_ (900 μatm), a transgenerational physiological buffering effect was observed at the highest *p*CO_2_ (1550 μatm). For the study presented here, we hypothesized that expression patterns of involved genes should follow these observations. To investigate this, we used an mRNA‐Seq approach, sequencing pools of individual copepods. We assembled and annotated a transcriptome using available arthropod sequences, and then searched for genes exhibiting changes in expression similar to the changes in fecundity. We also scanned all expressed sequences for single nucleotide polymorphism (SNP) frequency changes associated with exposure to the highest *p*CO_2_ treatment, to infer loci potentially under selection pressure. Finally, we searched for nonrandom functional annotations within genes exhibiting interesting expression patterns and changes in SNP frequencies. This combined approach allowed us to gain an increased understanding of the cellular targets of OA and of the relative importance of acclimation and adaptation. Using a physiological hypothesis‐testing strategy to mine gene expression data for co‐expression patterns, rather than traditional differential expression analyses, it was possible to increase the power to detect cellular functional targets of ocean acidification. This novel approach seems promising for future studies of effects of environmental changes in ecologically important nonmodel organisms, where long generation times and lack of replication is a constant issue.

## Materials and methods

### Experimental setup

*Pseudocalanus* spp. specimens were collected in the Gullmar fjord in the spring of 2013 (58°16′N, 11°26′E) using a 200 μm WP‐2 plankton net, after which they were kept in culture at the Sven Lovén Centre for Marine Sciences—Kristineberg in Fiskebäckskil, Sweden at 5°C. Species identity was confirmed through PCR with species‐specific primers: DNA from a pool of 100 indiv. was extracted and separated from RNA and proteins using TriZol reagent (Invitrogen). Primers used were for *P. minutus* PsCOI_1561F/COI_1931R; for *P. acuspes* PsCOI_1561F/COI_2060R (both described in Gudmundsdottir 2008); and for *P. elongatus* Pseud‐E 225‐27F/Pseud‐E 345‐22R (Grabbert et al. 2010). The PCR program used for *P. minutus* and *P. acuspes* was as follows: 94°C (45 s), 47°C (1 min), 72°C (1 min 30 s) for 40 cycles, and 72°C for 3 min; and the program used for *P. elongatus* was 94°C (1 min), 62°C (1 min), and 75°C (2 min) for 31 cycles.

After verifying presence of only *P. acuspes*, 200 adults (F_0_ generation) were transferred into each of three different *p*CO_2_ treatments: Control (400 μatm *p*CO_2_), Medium (900 μatm *p*CO_2_), and High (1550 μatm *p*CO_2_), with two replicates of each for a total of six laboratory populations. The laboratory populations were grown in 40‐L tanks with filtered seawater with a 12 h/12 h light/dark cycle at 5°C for 137 days, until the F_2_ generation reached maturity. pH was dynamically controlled using pH computers (Aqua Medic, Germany), applying the CO_2_ immediately next to the air flow. pH electrodes were placed inside the streams of bubbles. Total scale pH and total alkalinity were measured once a week, using a Metrohm 827 pH meter and by titration of 25 mL water in a SI Analytics Titroline potentiometric titrator (Riebesell et al. 2010), respectively, after which *p*CO_2_ was calculated in CO2sys version 1.4 (Lewis and Wallace 1998). For food, *Rhodomonas baltica* were pumped intermittently into the tanks to achieve satiating concentrations. Concentrations were measured every two days with an Elzone 5380 electronic particle counter. Water was changed in all tanks every two weeks by siphoning out water from a large 50‐μm sieve inserted into the tanks, then transferring the animals into clean tanks. Generation sorting (Both between the F_0_ and F_1_ generation and the F_1_–F_2_ generation) was achieved in a similar fashion during water change by manually removing all adult individuals after two weeks of egg production.

At maturation, the F_2_ adults were reciprocally transplanted: the 400 μatm laboratory populations (*n* = 2) were divided into thirds and placed in 400 μatm *p*CO_2_, 900 μatm *p*CO_2_, and 1550 μatm *p*CO_2_ conditions. The 900 μatm (*n* = 2) and 1550 μatm (*n* = 2) laboratory populations were split in halves: half placed back in their original *p*CO_2_ and half moved into 400 μatm *p*CO_2_ conditions. After three weeks, fecundity was measured (Thor and Dupont 2015), after which the animals were placed in RNAlater (Ambion, Foster City, CA, USA) at 4°C for 24 h, then frozen at −20°C for genetic analyses.

### Bioinformatic analysis and transcriptome assembly

RNA from 14 pools (seven treatments * two populations) of adult F_2_ copepods was extracted using TriZol reagent (Invitrogen, Carlsbad, CA, USA) (Table 1). Total RNA concentrations were measured using a NanoDrop 2000 spectrophotometer (Thermo Scientific, Waltham, MA, USA). For each pool, 1 μg of total RNA was used as input to Illumina TruSeq RNA sample kit v2 (Illumina, San Diego, CA, USA), following the kit standard protocol except in the final PCR step, where only 12 cycles were used rather than the recommended 15 to reduce the amount of duplicate sequences. Concentrations and fragment size distributions of the cDNA libraries were examined using a high‐sensitivity QuBit 2.0 fluorometric assay (Life Technologies, Carlsbad, CA, USA) and a TapeStation 2200 (Agilent, Santa Clara, CA, USA), respectively, after which libraries were combined equimolarly into three pools of four barcoded libraries each and one consisting of two libraries. Finally, all pools were diluted to 2 nm for sequencing. Sequencing was performed in February 2014 at the Genomics Core Facility of the University of Gothenburg, Sweden, in an Illumina NextSeq 500 sequencing machine with 50 bp read length, paired‐end sequencing.

**Table 1:** Sample size, number of reads, and alignment results from the 14 samples used in the study

Sample ID	Population	Sample size (*n* copepods)	*n* reads	% of reads aligning uniquely
1	400A	37	19 233 392	25.8
2	400B	68	22 531 143	23.1
3	900A	31	17 582 146	13.7
4	900B	50	13 269 154	9.5
5	1550A	39	21 055 372	22
6	1550B	49	21 006 096	24.1
7	400–900A	43	19 261 833	21.5
8	400–900B	58	20 218 284	25
9	400–1550A	38	20 271 884	16.4
10	400–1550B	28	22 877 758	23.4
11	900–400A	74	24 166 899	25.7
12	900–400B	76	20 619 393	25.7
13	1550–400A	68	12 020 876	8.7
14	1550–400B	57	24 491 817	23.4

The raw sequence data (Table 1) were processed on the University of Gothenburg computer cluster ‘Albiorix’. First, low‐quality (Q < 20) ends were trimmed, and adapter sequences were removed. Remaining sequence data were assembled into a transcriptome using Trinity (Grabherr et al. 2011) version r2013_08_14. Assembled contigs (*n* = 207 302) were annotated by 1. BLASTx against a BLAST database consisting of all arthropod sequences from the NCBI nr database (April 1, 2014); 2. BLASTx to the curated SwissProt database, using 10^−5^ as e‐value cutoff. From these searches, top hits were extracted as well as the nr top hit when omitting ‘putative’ and ‘hypothetical’ hits (as very often the descriptions of these hits are not very useful). In addition, GO and KEGG terms were extracted from the SwissProt BLAST results. To be conservative, all un‐annotated contigs were removed from the dataset, as these could potentially consist of contaminant sequences, for a final list of 69 555 annotated contigs. The quality trimmed sequence data were then aligned against the newly created transcriptome, keeping only reads aligning uniquely to one location.

### Gene expression analysis

**Figure 1:**
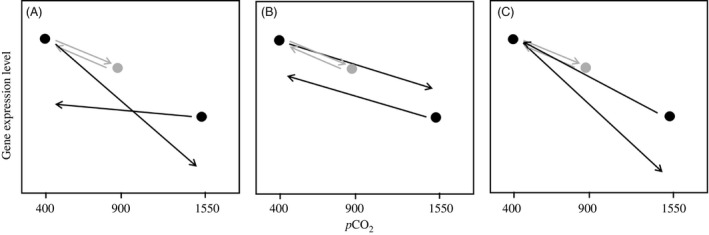
The three hypotheses used to examine the gene expression data. (A) Hypothesis 1: Different slopes but equal elevation interpreted as a transgenerational development of different reaction norms (i.e., transgenerational development of different phenotypic plasticity) at 1550 μatm *p*CO_2_; (B) Hypothesis 2: Equal slopes but different elevation interpreted as a transgenerational development of different phenotype (i.e., adaptation) at 1550 μatm *p*CO_2_; (C) Hypothesis 3: Different slopes and elevation. All three hypotheses assumed pure phenotypic plasticity between 400 and 900 μatm *p*CO_2_ as depicted by the gray arrows.

In cases where different sequences (‘seq’) of the same Trinity component (‘comp’) had the same annotation, it was concluded that they most likely represented different isoforms of the same gene. Thus, counts of different isoforms were combined into 28 879 ‘Unigene’ counts (mean *n* reads = 4.254 Mreads/sample, SD 1.645 Mreads). To compare gene expression levels between samples, counts were scale normalized in the DESeq package in R (Anders and Huber 2010). In addition, genes with greater standard deviation than mean and/or at least one sample with zero counts were excluded. A hypothesis‐testing method was employed to search for genes exhibiting a similar expression pattern as the observed changes in fecundity (see Figure 1 in Thor and Dupont 2015) using analysis of covariance (ancova using the SAS software (SAS Institute, Cary, NC, USA); the Shapiro–Wilk test was used to check that the data were normally distributed). Specifically, we scanned the expression data for genes showing no significant (*P *>* *0.05) difference in neither slope nor elevation (value of gene expression at the midpoint between the two treatments) of the linear regressions of the gene expression changes between the 400→900 and 900→400 μatm of *p*CO_2_ transplants (indicating phenotypic plasticity), AND:

1. significantly different slopes between the 400→1550 and 1550→400 μatm *p*CO_2_ transplant regression lines (‘Hypothesis 1’, i.e., transgenerational development of different phenotypic plasticity; Fig. 1A)
2. significantly different elevation between the 400→1550 and 1550→400 μatm *p*CO_2_ transplant regression lines (‘Hypothesis 2’, i.e., transgenerational development of different phenotype; Fig. 1B)
3. significantly different slopes and elevation between the 400→1550 and 1550→400 μatm *p*CO_2_ transplant regression lines (‘Hypothesis 3’, i.e., transgenerational development of both different phenotype and plasticity; Fig. 1C).

The lists of genes matching these three hypotheses were tested for nonrandom distribution of functions using a GO enrichment analysis with the online software called ‘Gene Ontology Enrichment Analysis Software Toolkit’ (GOEAST) (http://omicslab.genetics.ac.cn/GOEAST/) (Zheng and Wang 2008) in the ‘Custom Microarray’ setting, using the recommended settings (Hypergeometric tests with FDR under dependency).

**Figure 2:**
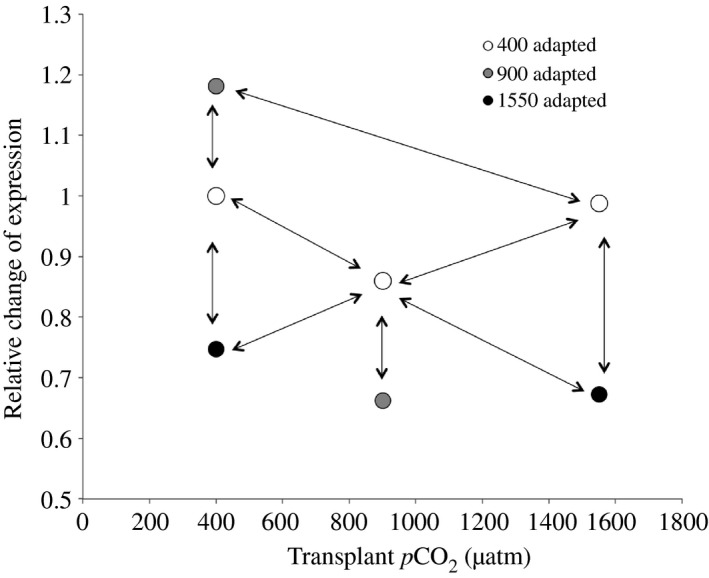
General expression pattern exhibited by contigs associated with helicase activity, used to scan the data for co‐expression patterns. Arrows indicate the logical rules used in the test.

As ‘Helicase activity’ was indicated as strongly overrepresented in the differential expression dataset matching Hypothesis 2 (see Results), we scanned the transcriptome for other genes exhibiting the same expression pattern, the idea being that anything with identical expression may be part of the same gene regulatory network. Means of the two replicates were calculated, and the logical test was designed as follows: (900→400 > 400→400 AND 400→400 > 1550→400 AND 400→900 > 900→900 AND 400→1550 > 1550→1550 AND 400→400 > 400→900 AND 400→1550 > 400→900 AND 400→900 > 1550→400 AND 400→900 > 1550→1550) (Fig. 2). The resulting list of genes passing these conditions was then tested for functional enrichment as described above.

### Allele frequency changes

The PoPoolation2 pipeline (Kofler et al. 2011) and scripts were used to analyze allele frequency changes in the pools, using the trimmed data files and only annotated contigs from the transcriptome assembly (although keeping isoforms separate, n_CONTIGS _= 69 555) (https://code.google.com/p/popoolation2/wiki/Tutorial), employing the SAMtools (Li et al. 2009) mpileup command to calculate allele frequencies at all sites for the 14 pools, then following with the Cochran–Mantel–Haenszel (CMH) test for testing for consistent and significant changes in allele frequency between the replicate treatments of 400 μatm *p*CO_2_ and 1550 μatm *p*CO_2_. A total of 747 423 variant sites identified by mpileup in the previous step were used for this test. We considered the 400→1550 μatm transplants as replicates of the 400 μatm treatments, and the 1550→400 μatm transplants as replicates of the 1550 μatm treatment, thus arriving at four replicates for the CMH test. As no mortality had occurred during the transplant, we assumed that allele frequencies had not changed during this time. A GO category functional enrichment test was conducted for the resulting gene list using GOEAST, as described above. In addition, gene expression levels between treatments were compared in these genes as well, as described above.

## Results

### Transcriptome assembly

The *Pseudocalanus acuspes* transcriptome assembly initially consisted of 207 302 contigs, with an N50 (contig length at which 50% of the assembly consists of contigs the same length or longer) of 851 bp, and a GC content of 51.1%. After removing contigs that could not be annotated, the remaining 69 555 contigs (Appendix S1) had an N50 of 1236 bp and a GC content of 53.3%, reflecting that shorter contigs are less likely to be annotated. Information about nr arthropod and SwissProt top hits, as well as GO and KEGG terms, is given in Appendix S2.

### Gene expression

**Figure 3:**
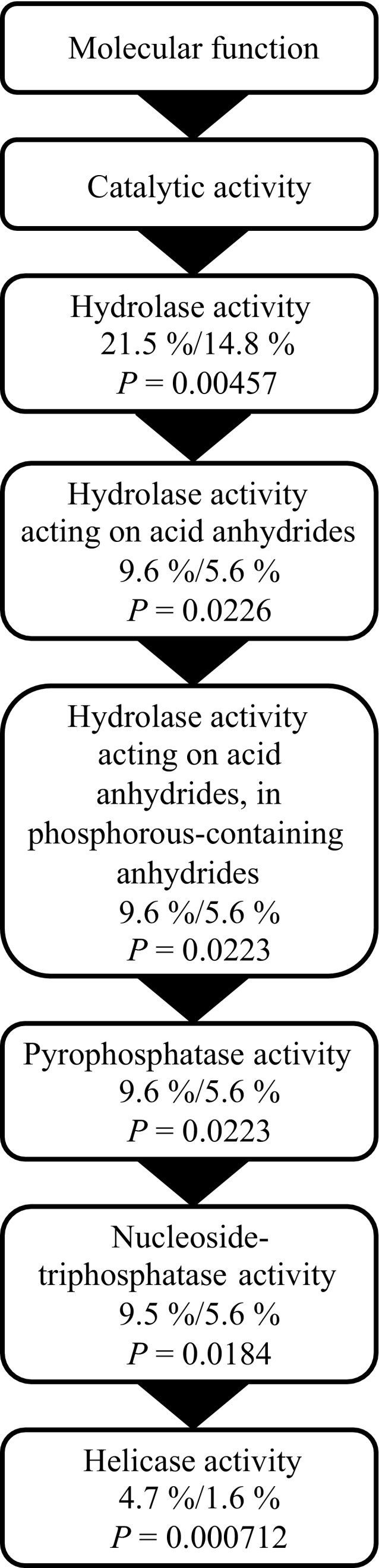
All Gene Ontology terms significantly enriched in the set of 686 contigs matching Hypothesis 2: Transgenerational development of different phenotype. Percentages are given as % contigs with GO term in list/% contigs with GO term in transcriptome, along with false‐discovery rate corrected *P*‐values.

**Figure 4:**
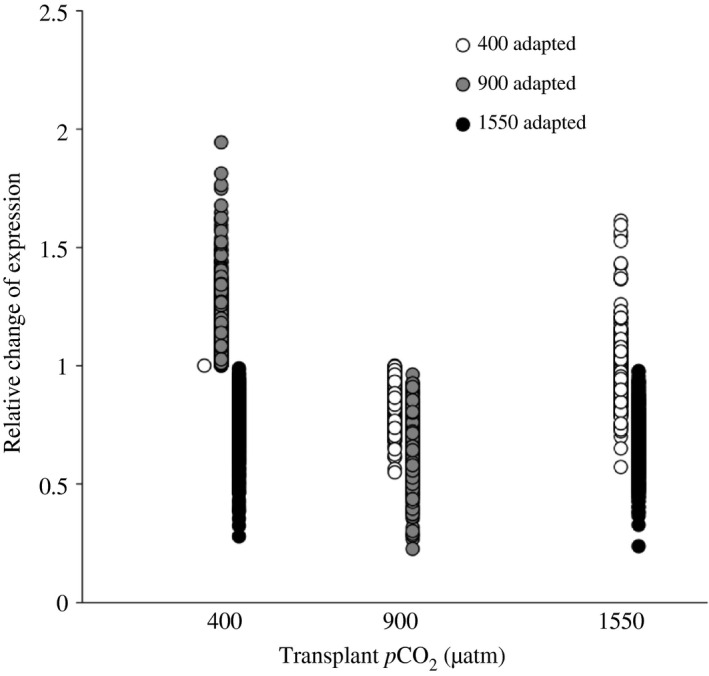
Expression of the 32 contigs associated with helicase activity. Expression levels are given as relative to the expression level in the control treatment.

Out of the 28 879 Unigenes, 15 850 remained after discarding ones with greater variance than mean and/or at least one sample with 0 counts after scaling normalization (Appendix S3). Out of these, 40.0% (6345 contigs) had a mean count across all samples >100, 41.5% (6570 contigs) had a mean count between 20 and 100, and 18.5% (2935 contigs) had a mean count < 20. While overall there was a large amount of variability between samples, both between and within treatments (see Figure S1), the ancova analysis identified 684 genes matching Hypothesis 1, 686 genes matching Hypothesis 2 and 26 genes matching Hypothesis 3 (see Fig. 1 for all hypotheses). The lists of genes matching Hypotheses 1 and 3 contained a random distribution of GO terms compared to the full transcriptomic dataset. However, the genes matching Hypothesis 2 were significantly enriched for a cascade of functions involving Helicase expression (Fig. 3). There were 32 genes involved in this functional category, all exhibiting the same pattern of decreased expression after spending two generations at 1550 μatm *p*CO_2_ (mean log twofold change −0.42; SD 0.20), even after being transplanted back into 400 μatm (mean log twofold change −0.42; SD 0.25) (Fig. 4). This reduction in expression, although not as severe, was also seen in both the short‐ and long‐term 900 μatm *p*CO_2_ treatments, but the populations that had been located in 900 μatm for two generations increased their expression back to the same expression level (or even slightly higher, although not significant) as the 400 μatm natives, when transplanted back into 400 μatm.

**Figure 5:**
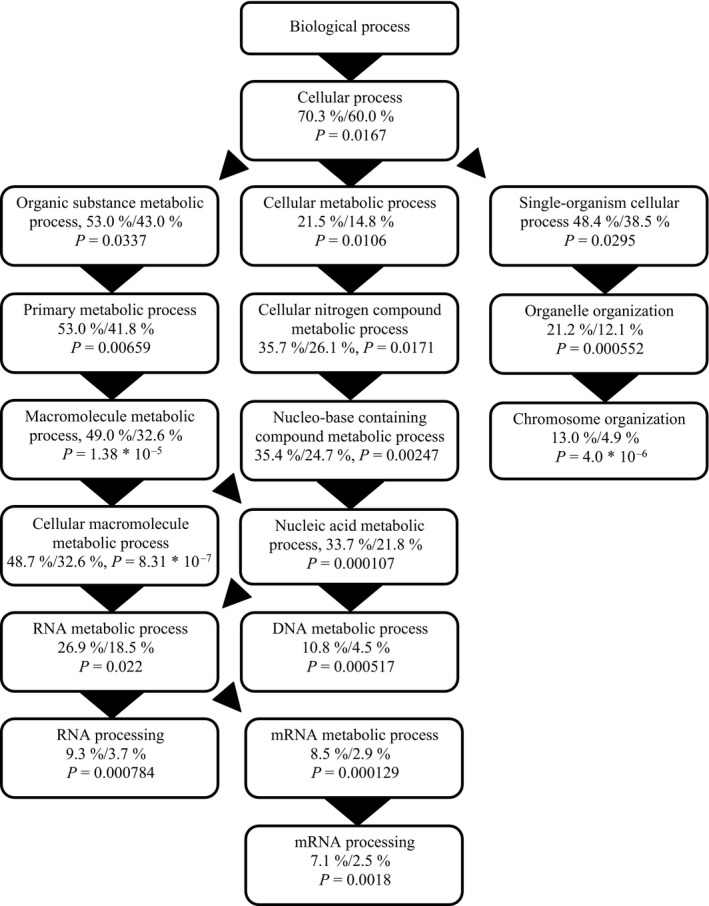
The most important Gene Ontology terms significantly enriched in the set of 353 contigs having the same expression pattern as the contigs involved in Helicase activity (for the full set, see Figure S2). Percentages are given as % contigs with GO term in list/% contigs with GO term in transcriptome, along with false‐discovery rate corrected *P*‐values.

Finally, we could also identify 321 additional contigs exhibiting the same expression pattern as the helicase contigs (Appendix S4), almost all of which are involved in RNA metabolism or DNA replication/repair (Fig. 5; Figure S2).

### Allele frequency changes

The SAMtools mpileup algorithm identified 747 423 polymorphic sites within the *P. acuspes* transcriptome. Within these, the CMH test identified 1517 SNPs and located in 303 contigs that were showing consistent and significant allele frequency changes among the biological replicates, after Bonferroni multiple‐test correction (*P *>* *0.05) (Appendices S5 and S6). These genes were strongly enriched for functions involving protein translation (GO:0006412, *P* = 4.43*10^−56^), proton transport (GO:0015985, *P* = 7.93*10^−7^), mitotic spindle elongat‐ion (GO:0000022, *P* = 5.24*10^−7^), and cellular respiration (mostly mitochondrial genes) (GO:0006754, *P* = 1.08*10^−5^) (see Figure S3 for all enriched terms). No significant gene expression changes between transplant treatments were seen in these genes, however.

## Discussion

In this study, we identified 303 genes that could be involved in adaptive evolution to *p*CO_2_ stress. This list included genes that encode for most of the proteins involved in ribosome formation and a large fraction of all mitochondrial genes. As all mitochondrial genes are inherited as one unit, it is difficult to speculate on which of these could be the ultimate target of natural selection, although it is interesting that also rather conserved sequences such as cytochrome oxidase subunit I also exhibit differences between treatments. Rather, it can be concluded that the mitochondrial function of oxidative phosphorylation was a target of natural selection. Respiration rates were higher at 900 μC, which was interpreted as a result of metabolic expenses due to an increased allocation of resources in order to acclimate to the new environment (Thor and Dupont 2015). Respiration rates were higher at 900 μatm as compared to 400 μatm and were correlated with a decrease in fecundity. As metabolic expenses vary depending on energy allocation to egg production in copepods (Thor 2002; Thor et al. 2002), Thor and Dupont (2015) interpreted this as a reallocation of resources caused by increased energy costs in high *p*CO_2_. On the contrary, at 1550 μatm respiration rates were similar to the ones at 400 μatm, with transplant tests suggesting that this could have arisen as a result of adaptation (Thor and Dupont 2015) due to differential mortality in the high *p*CO_2_ treatment. It therefore seems plausible that selection could be acting on maintenance of efficient oxidative phosphorylation at high *p*CO_2_ levels (Cortassa et al. 2009; Beaufort et al. 2011), to maintain energy production levels. We did not observe any increase in mitochondrial gene expression levels among treatments, which might have been expected if an increased energy demand had required more ATP to be produced. However, mitochondrial energy production can be modulated in more ways than expression levels (e.g., mtDNA copy number, transcription rate, mtRNA turnover, translation, mitochondrial fission/fusion). Other genes of interest showing allele frequency changes between treatments include Ferritin, which is associated with oxidative stress, known to be induced by high *p*CO_2_ levels in oysters (Tomanek et al. 2011), and also several subunits of the proteasome, which has been shown to evolve in response to high *p*CO2 stress in Sea Urchins (Pespeni et al. 2013).

A large fraction of the genes showing changes in allele frequencies between the high and low *p*CO_2_ treatments were mitochondrial, and must thus be linked as the mitochondrial genome is inherited maternally without recombination in copepods. Thus, the population of *P. acuspes* used in this experiment must have contained at least two mitochondrial lineages in order for this type of evolution to be observed. It is not uncommon for large marine invertebrate populations to show considerable variation in mitochondrial sequence (e.g., Silberman et al. 1994; Meyer and Paulay 2005), so this could be considered a realistic experimental situation. The rather rapid transfer of individuals (gradual increase over three days) from low to high *p*CO_2_ at the initiation of the experiment can be criticized as a less than realistic situation (although a frequent experimental practice), as ocean acidification is a slow and gradual process that will take place over a several hundred year period. By showing that *P. acuspes* has the potential to buffer even this rapid change, however, it is likely that they can do so also over longer time scales.

While genetic changes seem to be concentrated around the translational mechanism (ribosome formation) and mitochondrial functions, the gene expression data suggest transgenerational changes in RNA transcription and potentially DNA replication through changes in helicase activity. Interestingly, while the ancova approach used to identify genes with expression patterns matching the observed changes in fecundity only used ‘difference in expression elevation’ (between acute versus multigeneration treatment in 1550 μatm) as a criterion without considering the direction of the change (up‐ or down‐regulation), all of the 32 contigs associated with helicase activity showed the same pattern (Fig. 4): Strong down‐regulation after two generations at 1550 μatm *p*CO_2_, with no compensation when transplanted back into 400 μatm *p*CO_2_. At the intermediate 900 μatm *p*CO_2_, a decrease in expression (although not as strong as at 1550 μatm *p*CO_2_) can be seen at both acute and transgenerational scales, but upon reintroduction into 400 μatm *p*CO_2_ expression re‐attained 400 μatm levels. These changes in expression could be associated with changes in energy allocation to different functions.

All contigs associated with helicase activity consistently showed identical expression patterns (Fig. 2) despite being identified though ancova by the rather vague ‘Difference in elevation’ approach (‘Hypothesis 2’). Thus, it was of interest to scan the gene expression data for contigs showing the same pattern, to potentially identify other genes part of the same regulatory network. This search identified 353 contigs (including the helicase contigs) involved in RNA transcription and DNA replication (Fig. 5, Appendix S4). We cannot distinguish which cellular functions are the primary targets of this consistent down‐regulation of transcription/replication, but rather conclude that this can play a role in changes in energy allocation to different functions.

Using both the gene expression and the SNP frequency change data, a picture emerges on the cellular response and adaptive potential of *P. acuspes* to *p*CO_2_ stress. The high *p*CO_2_ level induces a transgenerational change in helicase activity [either through natural selection in a control region (Wray 2007), or nongenetic changes (Goldberg et al. 2007)], which is used to modify energy allocation. Helicase is involved in the separation of double‐stranded DNA and is a regulator of both RNA transcription and DNA replication, so changes in helicase expression would have significant effects on certain cellular functions, depending on the type of helicase involved. For example, reducing costs associated to DNA replication/cellular division, thereby reducing egg production as observed, would allow energy to be allocated to maintenance of homeostasis through ion pumps. Interestingly, a similar pattern has been observed in sea urchins, where gonadal tissue was used as an energy source during acclimation to pH changes (Dupont et al. 2013). At the same time, there is a selective advantage (either through selective mortality or perhaps more likely through larval development) for certain ribosomal structures and mitochondria that are better suited to a low‐energy metabolic mode.

From the results of this study, we cannot conclude which specific physiological functions are down‐regulated and what the long‐term fitness consequences might be (apart from the apparent decrease in fecundity). For example, elevated *p*CO_2_ can lead to modulation of the immune‐response and lead to a reduced ability to fight against pathogens (e.g., Asplund et al. 2014), and reduced levels of genetic variability due to one selective factor might impede the population's ability to adapt to additional stressors (Pistevos et al. 2011). Thus, an exciting field for future studies lies within investigating consequences of the observed ‘adaptation’ in the context of multiple environmental drivers (Dupont and Pörtner 2013). Despite these issues, this study demonstrates the great ability that marine invertebrates have to adapt from standing genetic variation. In most cases, these small organisms harbor large amounts of genetic diversity and have large population sizes and short generation times (Hellberg et al. 2002), so adaptation from standing genetic variation will surely have a large role in the maintenance of ecosystem stability in an unstable future environment.

Presently, the long‐term consequences of ocean acidification are difficult to predict. Even between closely related taxa, short‐term responses vary considerably (see, e.g., Kroeker et al. 2010), and very little is known about the effects of evolutionary change on a global scale. However, by understanding the effects of OA from a cellular perspective over a longer timeframe in ecologically important species (such as copepods), we might be able to build predictive models of global ecosystem changes in the future. To do this, much more data will be needed from a variety of different organisms, but as sequencing methods become more available and easier to use and standards for experimental design are being improved, this goal becomes more and more achievable in the years to come. In addition, by testing gene expression data against specific hypotheses generated by physiological data, we can gain power in detecting the cellular mechanisms involved in adaptation and acclimation to OA, and we predict that future studies increasingly will adopt this type of approach rather than the more exploratory differential gene expression analyses used to date.

## Data archiving statement

All raw Illumina reads have been submitted to the NCBI Short Read Archive (SRA) (Bioproject SRP063962). The transcriptome assembly and annotation, as well as gene expression count data, are available as online supporting material (Appendices S1–S3).

## Supporting information


**Appendix S1**. *De novo* transcriptome assembly of *P. acuspes*.

**Appendix S2.** Annotation data for all assembly contigs.

**Appendix S3.** Scale‐normalized count data.

**Appendix S4.** List of contigs exhibiting the same expression pattern as the helicase‐annotated contigs.

**Appendix S5.** List of SNPs with significant results from the CMH‐test.

**Appendix S6.** Annotation of contigs containing SNPs with significant CMH‐test results.

**Figure S1** PCA plot of the 2 most informative dimensions of the gene expression data.

**Figure S2.** GO terms significantly overrepresented in the list of contigs matching the expression pattern observed in the helicase‐annotated contigs (Appendix S4).

**Figure S3** GO terms significantly overrepresented in the list of contigs containing SNPs with significant CMH‐test results (Appendix S6).
