# Supplementary figures and images for: Selection on oxidative phosphorylation and ribosomal structure as a multigenerational response to ocean acidification in the common copepod Pseudocalanus acuspes
Source: Evol Appl. 2015 Nov 23;9(9):1112–23. doi: 10.1111/eva.12335 (PMC5039324; doi:10.1111/eva.12335)

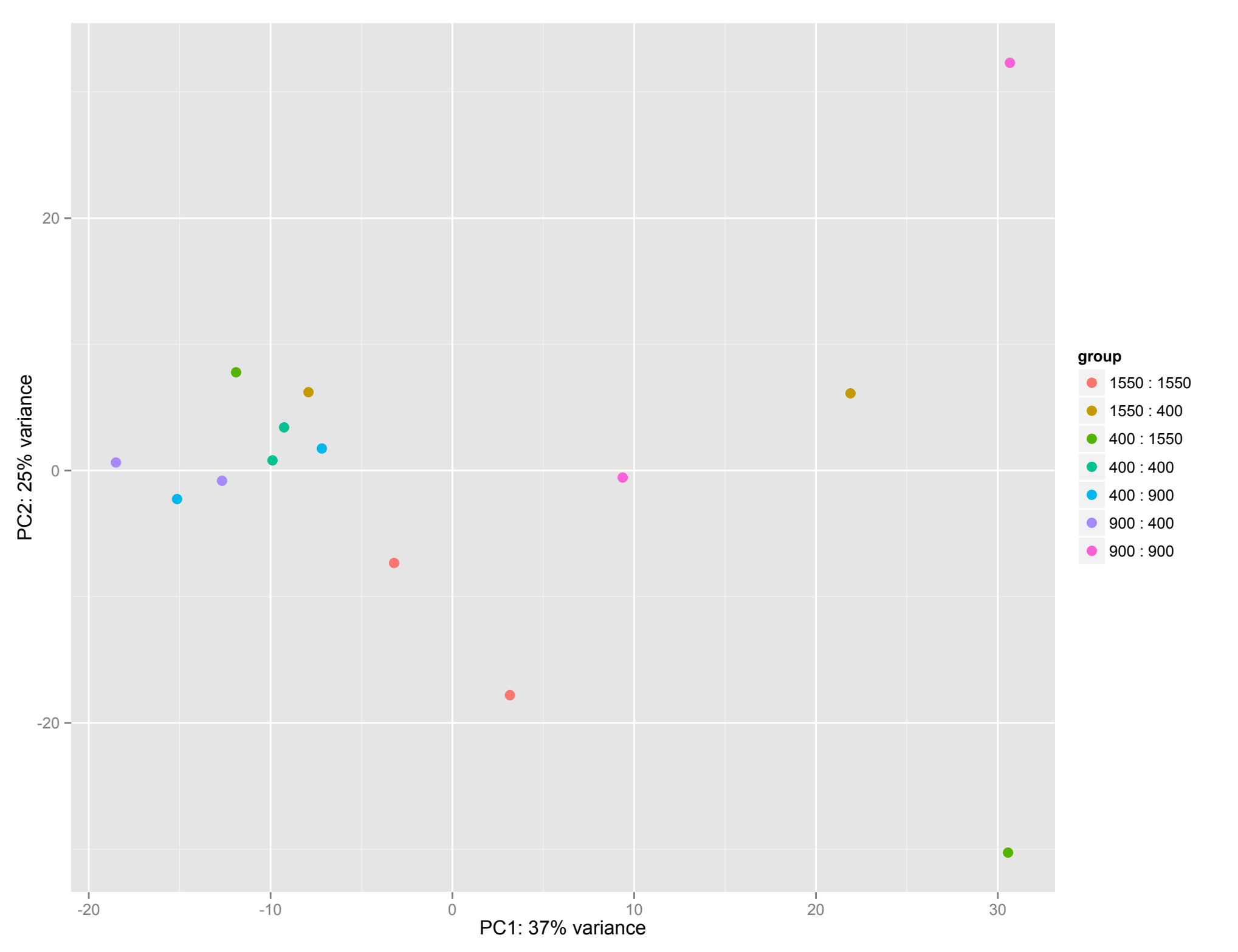

Supplement: Supplementary file 7 — Figure S1 PCA plot of the 2 most informative dimensions of the gene expression data. [file EVA-9-1112-s007.pdf]

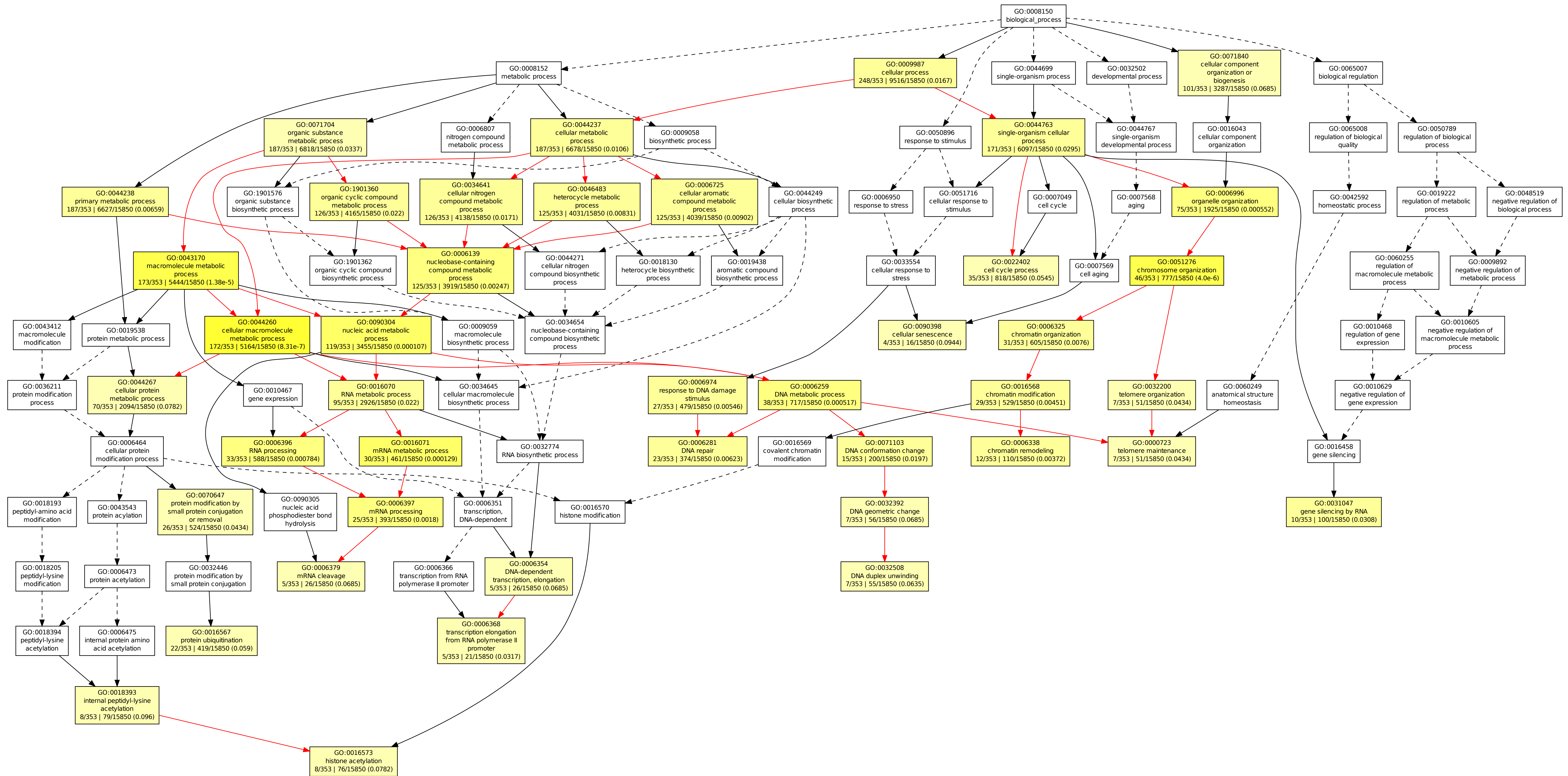

Supplement: Supplementary file 8 — Figure S2. GO terms significantly overrepresented in the list of contigs matching the expression pattern observed in the helicase‐annotated contigs (Appendix S4). [file EVA-9-1112-s008.pdf]
