# Supplementary material for: Selection on oxidative phosphorylation and ribosomal structure as a multigenerational response to ocean acidification in the common copepod Pseudocalanus acuspes
Source: Evol Appl. 2015 Nov 23;9(9):1112–23. doi: 10.1111/eva.12335 (PMC5039324; doi:10.1111/eva.12335)
Supplement: Supplementary file 9 — Figure S3 GO terms significantly overrepresented in the list of contigs containing SNPs with significant CMH‐test results (Appendix S6). [file EVA-9-1112-s009.pdf]

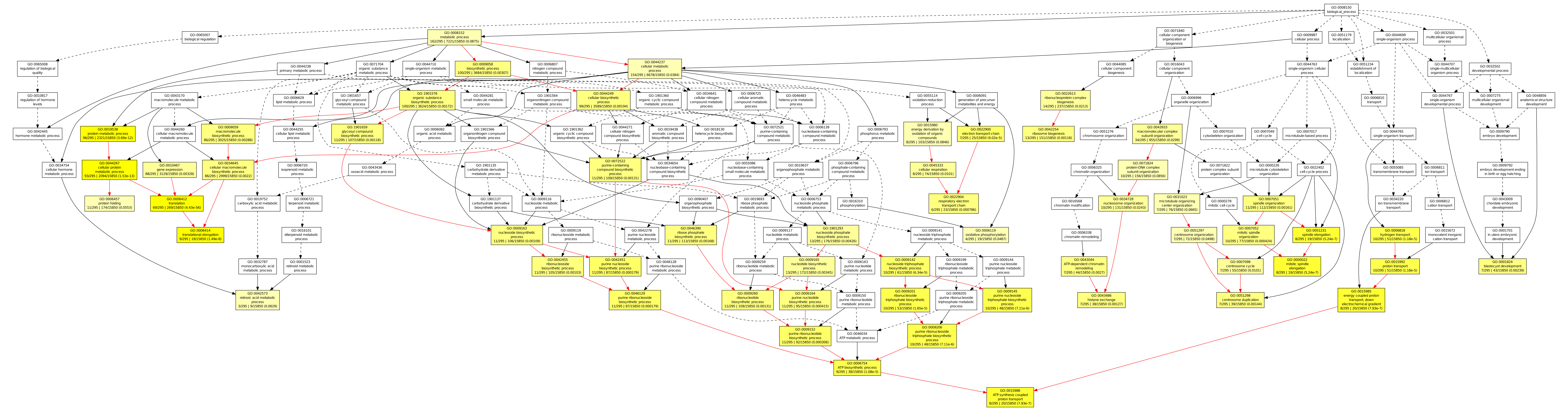
GO:0008150 biological_process
GO:0065007 biological regulation
GO:0008152 metabolic process 162/295 | 7221/15850 (0.0875)
GO:0071840 cellular component organization or biogenesis
GO:0009987 cellular process
GO:0051179 localization
GO:0044699 single-organism process
GO:0032501 multicellular organismal process
GO:0065008 regulation of biological quality
GO:0044238 primary metabolic process
GO:0071704 organic substance metabolic process
GO:0044710 single-organism metabolic process
GO:0009058 biosynthetic process 100/295 | 3684/15850 (0.00307)
GO:0006807 nitrogen compound metabolic process
GO:0044237 cellular metabolic process 154/295 | 6678/15850 (0.0384)
GO:0044085 cellular component biogenesis
GO:0016043 cellular component organization
GO:0044763 single-organism cellular process
GO:0051234 establishment of localization
GO:0044707 single-multicellular organism process
GO:0032502 developmental process
GO:0010817 regulation of hormone levels
GO:0043170 macromolecule metabolic process
GO:0006629 lipid metabolic process
GO:1901657 glycosyl compound metabolic process
GO:1901576 organic substance biosynthetic process 100/295 | 3624/15850 (0.00172)
GO:0044281 small molecule metabolic process
GO:1901564 organonitrogen compound metabolic process
GO:0044249 cellular biosynthetic process 99/295 | 3589/15850 (0.00194)
GO:1901360 organic cyclic compound metabolic process
GO:0034641 cellular nitrogen compound metabolic process
GO:0006725 cellular aromatic compound metabolic process
GO:0046483 heterocycle metabolic process
GO:0055114 oxidation-reduction process
GO:0006091 generation of precursor metabolites and energy
GO:0022613 ribonucleoprotein complex biogenesis 14/295 | 237/15850 (0.0213)
GO:0006996 organelle organization
GO:0006810 transport
GO:0044767 single-organism developmental process
GO:0007275 multicellular organismal development
GO:0048856 anatomical structure development
GO:0042445 hormone metabolic process
GO:0019538 protein metabolic process 96/295 | 2321/15850 (3.69e-12)
GO:0044260 cellular macromolecule metabolic process
GO:0009059 macromolecule biosynthetic process 86/295 | 3025/15850 (0.00288)
GO:0044255 cellular lipid metabolic process
GO:1901659 glycosyl compound biosynthetic process 11/295 | 107/15850 (0.00118)
GO:0006082 organic acid metabolic process
GO:1901566 organonitrogen compound biosynthetic process
GO:1901362 organic cyclic compound biosynthetic process
GO:0044271 cellular nitrogen compound biosynthetic process
GO:0019438 aromatic compound biosynthetic process
GO:0018130 heterocycle biosynthetic process
GO:0072521 purine-containing compound metabolic process
GO:0006139 nucleobase-containing compound metabolic process
GO:0006793 phosphorus metabolic process
GO:0015980 energy derivation by oxidation of organic compounds 8/295 | 103/15850 (0.0846)
GO:0022900 electron transport chain 7/295 | 25/15850 (9.02e-5)
GO:0042254 ribosome biogenesis 13/295 | 151/15850 (0.00118)
GO:0051276 chromosome organization
GO:0043933 macromolecular complex subunit organization 34/295 | 955/15850 (0.0299)
GO:0007010 cytoskeleton organization
GO:0007049 cell cycle
GO:0007017 microtubule-based process
GO:0044765 single-organism transport
GO:0009790 embryo development
GO:0034754 cellular hormone metabolic process
GO:0044267 cellular protein metabolic process 93/295 | 2094/15850 (1.53e-13)
GO:0010467 gene expression 88/295 | 3129/15850 (0.00328)
GO:0034645 cellular macromolecule biosynthetic process 86/295 | 2999/15850 (0.0022)
GO:0006720 isoprenoid metabolic process
GO:0043436 oxoacid metabolic process
GO:1901135 carbohydrate derivative metabolic process
GO:0072522 purine-containing compound biosynthetic process 11/295 | 109/15850 (0.00131)
GO:0034654 nucleobase-containing compound biosynthetic process
GO:0055086 nucleobase-containing small molecule metabolic process
GO:0019637 organophosphate metabolic process
GO:0006796 phosphate-containing compound metabolic process
GO:0045333 cellular respiration 8/295 | 74/15850 (0.0101)
GO:0006325 chromatin organization
GO:0071824 protein-DNA complex subunit organization 10/295 | 156/15850 (0.0856)
GO:0071822 protein complex subunit organization
GO:0000226 microtubule cytoskeleton organization
GO:0022402 cell cycle process
GO:0055085 transmembrane transport
GO:0006811 ion transport
GO:0009792 embryo development ending in birth or egg hatching
GO:0006457 protein folding 11/295 | 174/15850 (0.0553)
GO:0006412 translation 69/295 | 269/15850 (4.43e-56)
GO:0019752 carboxylic acid metabolic process
GO:0006721 terpenoid metabolic process
GO:1901137 carbohydrate derivative biosynthetic process
GO:0009116 nucleoside metabolic process
GO:0090407 organophosphate biosynthetic process
GO:0019693 ribose phosphate metabolic process
GO:0006753 nucleoside phosphate metabolic process
GO:0016310 phosphorylation
GO:0022904 respiratory electron transport chain 6/295 | 23/15850 (0.000786)
GO:0016568 chromatin modification
GO:0034728 nucleosome organization 10/295 | 131/15850 (0.0243)
GO:0031023 microtubule organizing center organization 7/295 | 76/15850 (0.0665)
GO:0000278 mitotic cell cycle
GO:0007051 spindle organization 11/295 | 112/15850 (0.00161)
GO:0034220 ion transmembrane transport
GO:0006812 cation transport
GO:0043009 chordate embryonic development
GO:0006414 translational elongation 9/295 | 19/15850 (1.49e-8)
GO:0016101 diterpenoid metabolic process
GO:0009163 nucleoside biosynthetic process 11/295 | 106/15850 (0.00109)
GO:0009119 ribonucleoside metabolic process
GO:0042278 purine nucleoside metabolic process
GO:0046390 ribose phosphate biosynthetic process 11/295 | 113/15850 (0.00168)
GO:1901293 nucleoside phosphate biosynthetic process 13/295 | 176/15850 (0.00426)
GO:0009117 nucleotide metabolic process
GO:0009141 nucleoside triphosphate metabolic process
GO:0006119 oxidative phosphorylation 4/295 | 19/15850 (0.0487)
GO:0006338 chromatin remodeling
GO:0051297 centrosome organization 7/295 | 72/15850 (0.0498)
GO:0007052 mitotic spindle organization 10/295 | 77/15850 (0.000424)
GO:0051231 spindle elongation 8/295 | 19/15850 (5.24e-7)
GO:0006818 hydrogen transport 10/295 | 51/15850 (1.18e-5)
GO:0015672 monovalent inorganic cation transport
GO:0001701 in utero embryonic development
GO:0032787 monocarboxylic acid metabolic process
GO:0001523 retinoid metabolic process
GO:0042455 ribonucleoside biosynthetic process 11/295 | 105/15850 (0.00103)
GO:0042451 purine nucleoside biosynthetic process 11/295 | 87/15850 (0.000179)
GO:0046128 purine ribonucleoside metabolic process
GO:0009259 ribonucleotide metabolic process
GO:0009165 nucleotide biosynthetic process 13/295 | 172/15850 (0.00345)
GO:0006163 purine nucleotide metabolic process
GO:0009142 nucleoside triphosphate biosynthetic process 10/295 | 61/15850 (6.34e-5)
GO:0009199 ribonucleoside triphosphate metabolic process
GO:0009144 purine nucleoside triphosphate metabolic process
GO:0043044 ATP-dependent chromatin remodeling 7/295 | 44/15850 (0.0027)
GO:0007098 centrosome cycle 7/295 | 55/15850 (0.0101)
GO:0000022 mitotic spindle elongation 8/295 | 19/15850 (5.24e-7)
GO:0015992 proton transport 10/295 | 51/15850 (1.18e-5)
GO:0001824 blastocyst development 7/295 | 43/15850 (0.00239)
GO:0042573 retinoic acid metabolic process 3/295 | 9/15850 (0.0629)
GO:0046129 purine ribonucleoside biosynthetic process 11/295 | 87/15850 (0.000179)
GO:0009260 ribonucleotide biosynthetic process 11/295 | 109/15850 (0.00131)
GO:0006164 purine nucleotide biosynthetic process 11/295 | 95/15850 (0.000415)
GO:0009150 purine ribonucleotide metabolic process
GO:0009201 ribonucleoside triphosphate biosynthetic process 10/295 | 53/15850 (1.65e-5)
GO:0009205 purine ribonucleoside triphosphate metabolic process
GO:0009145 purine nucleoside triphosphate biosynthetic process 10/295 | 48/15850 (7.11e-6)
GO:0043486 histone exchange 7/295 | 38/15850 (0.00127)
GO:0051298 centrosome duplication 7/295 | 39/15850 (0.00144)
GO:0015985 energy coupled proton transport, down electrochemical gradient 8/295 | 20/15850 (7.93e-7)
GO:0009152 purine ribonucleotide biosynthetic process 11/295 | 92/15850 (0.000306)
GO:0046034 ATP metabolic process
GO:0009206 purine ribonucleoside triphosphate biosynthetic process 10/295 | 48/15850 (7.11e-6)
GO:0006754 ATP biosynthetic process 9/295 | 38/15850 (1.08e-5)
GO:0015986 ATP synthesis coupled proton transport 8/295 | 20/15850 (7.93e-7)
